# Immunomodulating Profile of Dental Mesenchymal Stromal Cells: A Comprehensive Overview

**DOI:** 10.3389/froh.2021.635055

**Published:** 2021-03-31

**Authors:** Alessia Paganelli, Oriana Trubiani, Francesca Diomede, Alessandra Pisciotta, Roberto Paganelli

**Affiliations:** ^1^PhD Program in Clinical and Experimental Medicine, University of Modena and Reggio Emilia, Modena, Italy; ^2^Surgical, Medical and Dental Department of Morphological Sciences Related to Transplant, Oncology and Regenerative Medicine, University of Modena and Reggio Emilia, Modena, Italy; ^3^Department of Medical, Oral and Biotechnological Sciences, University “G. D'Annunzio” Chieti-Pescara, Chieti, Italy; ^4^Department of Medicine and Aging Sciences, University “G. D'Annunzio” Chieti-Pescara, Chieti, Italy; ^5^YDA, Institute of Clinical Immunotherapy and Advanced Biological Treatments, Pescara, Italy

**Keywords:** T cells, immunomodulation, dental, cytokines, extracellular vesicles, mesenchymal stem cells (MeSH ID D059630)

## Abstract

Dental mesenchymal stromal cells (MSCs) are multipotent cells present in dental tissues, characterized by plastic adherence in culture and specific surface markers (CD105, CD73, CD90, STRO-1, CD106, and CD146), common to all other MSC subtypes. Dental pulp, periodontal ligament, apical papilla, human exfoliated deciduous teeth, alveolar bone, dental follicle, tooth germ, and gingiva are all different sources for isolation and expansion of MSCs. Dental MSCs have regenerative and immunomodulatory properties; they are scarcely immunogenic but actively modulate T cell reactivity. *in vitro* studies and animal models of autoimmune diseases have provided evidence for the suppressive effects of dental MSCs on peripheral blood mononuclear cell proliferation, clearance of apoptotic cells, and promotion of a shift in the Treg/Th17 cell ratio. Appropriately stimulated MSCs produce anti-inflammatory mediators, such as transforming growth factor-β (TGF-β), prostaglandin E2, and interleukin (IL)-10. A particular mechanism through which MSCs exert their immunomodulatory action is *via* the production of extracellular vesicles containing such anti-inflammatory mediators. Recent studies demonstrated MSC-mediated inhibitory effects both on monocytes and activated macrophages, promoting their polarization to an anti-inflammatory M2-phenotype. A growing number of trials focusing on MSCs to treat autoimmune and inflammatory conditions are ongoing, but very few use dental tissue as a cellular source. Recent results suggest that dental MSCs are a promising therapeutic tool for immune-mediated disorders. However, the exact mechanisms responsible for dental MSC-mediated immunosuppression remain to be clarified, and impairment of dental MSCs immunosuppressive function in inflammatory conditions and aging must be assessed before considering autologous MSCs or their secreted vesicles for therapeutic purposes.

## Introduction

Mesenchymal stromal cells (MSCs) are a subset of multipotent cells present in tissues of mesenchymal origin, mainly responsible for their regeneration. MSCs were first identified as a specific subset of spindle-shaped cells in the bone marrow, characterized by adherence to plastic under standard culture conditions, with the potential for clonogenic proliferation. In 2006, the Mesenchymal and Tissue Stem Cell Committee of the International Society for Cellular Therapy defined three minimal criteria for MSCs: plastic adherence, ability to differentiate into chondroblasts, osteoblasts, and adipocytes *in vitro*, and the presence of several specific surface markers, such as CD105, CD73, and CD90 [1]. More recently, the nomenclature has been revised, and novel specific surface molecules have been identified: MSCs are now defined also as STRO-1, CD106, and CD146 positive cells [2–5].

After the isolation and characterization of bone marrow stromal stem cells (BMSCs), other MSC-like populations have been identified in other tissues and organs [6, 7]. MSC sources include umbilical-cord, amniotic-fluid, adipose, and dental tissue [8–10]. Similar MSC populations can also be found in skeletal muscle, synovium, liver, lungs, tendons, placenta, dermis, and breast milk [11–15].

All MSC subpopulations not only share self-renewal capabilities and multipotency but also display immunomodulatory properties [16, 17].

With regard to dental tissue-derived MSCs, eight different subsets of MSCs have been identified so far: dental-pulp MSCs (DPMSCs), periodontal-ligament MSCs (PDLMSCs), MSCs from apical papilla (MSCAPs), MSCs from human exfoliated deciduous teeth (MSCHEDTs), alveolar bone-derived MSCs (ABMSCs), dental follicle progenitor cells (DFPCs), tooth germ progenitor cells (TGPCs), and gingival MSCs (GMSCs) [18–20]. MSCs derived from the oral cavity are particularly interesting in terms of their embryogenesis in that dental MSCs originate from migrating neural crest cells in the lateral ridges of the neural plate [21]. Neural crest cells possess stemness and multipotency features, play a strategic role in tooth organ development, and contribute to craniofacial bone formation [22].

**Figure 1:**
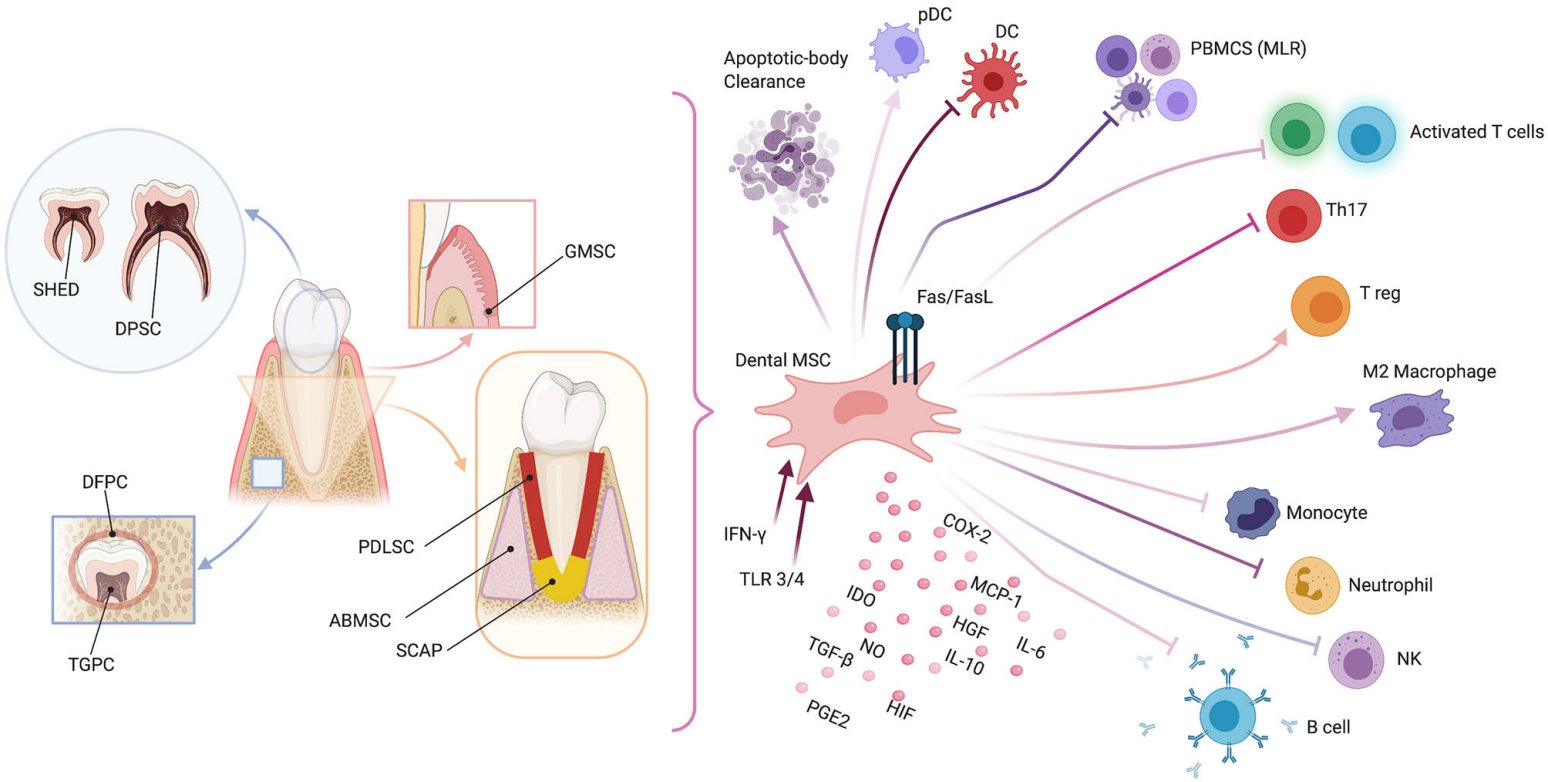
Schematic representation of dental mesenchymal stromal cell (MSC) sources (left panel) and possible immunomodulating mechanisms (right panel). Dental pulp MSCs (DPMSCs), MSCs from human exfoliated deciduous teeth (MSCHEDTs), periodontal ligament MSCs (PDLMSCs), alveolar-bone derived MSCs (ABMSCs), gingival MSCs (GMSCs), MSCs from apical papilla (MSCAPs), dental follicle progenitor cells (DFPCs), and tooth germ progenitor cells (TGPCs). Peripheral blood mononucleated cells (PBMCS), mixed lymphocyte reaction (MLR), plasmacytoid dendritic cell (pDC), natural killer (NK), type-2 cyclooxygenase (COX2), Fas ligand (FasL), indoleamine 2,3-dioxygenase (IDO), nitric oxide (NO), transforming growth factor-β (TGF-β), hepatocyte growth factor (HGF), prostaglandin E2 (PGE2), hypoxia-induced factor (HIF), interleukins 6 and 10 (IL6 and IL10, respectively), monocyte chemoattractant protein-1 (MCP-1), Toll-like receptor (TLR), and interferon-γ (IFN-γ). Created with BioRender.com.

As for other types of MSCs, dental MSCs are currently widely studied for their immune properties [23]. Here, we briefly describe the immunomodulating properties typical for each subset of MSCs (see Figure 1).

## Dental Pulp Mesenchymal Stromal Cells

DPMSCs were the first human dental MSCs identified in 2000 by Gronthos et al. [24]. DPMSCs are today widely used in clinical trials for regenerative purposes. Many groups already demonstrated that DPMSCs are capable of T cell inhibition and therefore have the potential for modulating T cell reactivity associated with both autoimmune diseases and allogeneic tissue transplantation [25]. Inhibition of peripheral blood mononuclear cell proliferation *in vitro* is thought to occur *via* the production of soluble factors secreted by DPMSCs, induced by interferon (IFN)-γ. The immunosuppressive effect of DPMSCs was alternatively shown to be triggered by activation of Toll-like receptors (TLRs) through the upregulation of specific cytokines and growth factors, such as IL-6 and TGF-β [26]. In addition, DPMSCs can induce apoptosis of activated T cells *via* direct cell-to-cell interactions, mediated by the Fas ligand [27]. DPMSCs also interact with activated neutrophils: a recent article demonstrated enhanced IFN-γ and IL-6 production after coculturing. Moreover, rapid and significant commitment toward the osteogenic lineage is achieved by neutrophil-exposed DPMSCs [28]. DPMSCs' immunomodulatory ability was deeply investigated by Martinez and co-authors in *in vitro*-induced hypoxic conditions. DPMSCs were not only shown to dampen dendritic cell (DC) differentiation from monocytes but also efficiently recruited monocytes with immunosuppressive potential, as demonstrated by the M2-phenotype of macrophages and high levels of IL-10. Moreover, DPMSCs were demonstrated both to determine impairment in natural killer (NK) degranulation and to have enhanced resistance to NK cell-mediated lysis. Lastly, DPMSCs' pro-angiogenic properties were also described [29]. Several authors have hypothesized the presence of different subpopulations with different activity among DPMSCs [30]; whether the immunosuppressive phenotype strictly correlates with the presence of specific surface markers still needs to be determined.

## Mesenchymal Stromal Cells from Human Exfoliated Deciduous Teeth

MSCHEDTs are the DPMSC's counterpart in deciduous teeth, discovered in 2003 by Miura et al. [31]. MSCHEDTs significantly inhibit the differentiation of the pro-inflammatory subset of T helper 17 (Th17) cells and promote the induction of regulatory T cells (Tregs) *ex vivo*, being even more efficient than BMSCs for Th17 inhibition [32]. Their immunomodulatory effect has already been demonstrated in canine models of muscular dystrophy [33].

In murine models, systemic infusion of MSCHEDTs was able to effectively reverse systemic lupus erythematosus (SLE)-associated manifestations, probably because of a shift in the Treg/Th17 cell ratio. Potentially, their efficacy in SLE models could also be due to clearance of apoptotic cells by MSCHEDTs, as already demonstrated for other types of MSCs [34].

## Periodontal Ligament Mesenchymal Stromal Cells

PDLMSCs were isolated and described in detail for the first time by Seo et al. [35] and Trubiani et al. [36]. PDLMSCs, similar to other MSCs of different origins, are sensitive to specific stimuli. One such stimulus for the expression of immunomodulatory properties is a coculture with peripheral blood mononuclear cells and specific cytokines such as IFN-γ [37, 38]. After *in vitro* exposure to IFN-γ, the expression of hepatocyte growth factor, indoleamine 2,3-dioxygenase (IDO), and TGF-β was upregulated, leading to immunosuppression [39]. PDLMSCs were also shown to induce T cell anergy through the secretion of prostaglandin E2 (PGE2) [40].

In animal models of experimental autoimmune encephalomyelitis, decreased signs of inflammation and demyelination in the spinal cord are observed after injection of PDLMSCs, both through the increased production of neurotrophic factors and the suppression of inflammatory mediators [41]. The cell-conditioned medium reduced inflammatory damage in the same model and purified extracellular vesicles from PDLMSCs that mediated similar effects [42]. The vesicles were found to contain the anti-inflammatory cytokines IL-10 and TGF-β. However, PDLMSCs from inflamed periodontium were shown recently to have significantly diminished inhibitory effects on T cell proliferation, compared with cells from healthy tissue, mainly due to a reduced induction of Tregs [43]. These findings may be relevant to the pathogenesis of periodontitis and should direct the efforts toward developing therapeutics for periodontitis by exploiting immunomodulation.

## Mesenchymal Stromal Cells of Apical Papilla

The apical papilla is the part of the soft tissue found at the apex of developing teeth. MSCAPs were discovered in human immature permanent teeth in 2006 by Sonoyama et al. [44, 45].

Relative to DPMSCs, MSCAPs show higher proliferation rates and mediate more efficient regeneration of the dentin matrix. Thus, developing dental tissues are probably a better source of immature stromal cells. MSCAPs are scarcely immunogenic and inhibit mixed lymphocyte reactions mainly through the secretion of soluble factors [46]. Conveniently, cryopreservation does not seem to alter MSCAPs' immune properties [47].

## Alveolar Bone Mesenchymal Stromal Cells

Recently, a unique population of MSCs referred to as ABMSCs has been isolated from the alveolar bone [48]. The isolation procedure is considered particularly easy and feasible when performed during implant positioning. These cells morphologically and functionally resemble the other types of dental MSCs described. Very recent studies confirmed *in vitro* ABMSCs' immunosuppressive effects both on monocyte and T cell activation. Moreover, ABMS was found to induce polarization of macrophages toward an anti-inflammatory phenotype (M2) and was able to secrete IL-6 and MCP-1 [49].

## Dental Follicle Precursor Cells

The dental follicle (DF) is a vascular fibrous sac containing the developing tooth and its odontogenic organ before eruption [50]. The DF eventually differentiates into the periodontal ligament. A subset of progenitor cells with the characteristics of MSCs was isolated from the DF of human third molars in 2005 by Morsczeck et al. using the same protocol as Gronthos et al. used before for DPMSCs [51]. Recent studies have shown that DFPCs can inhibit the mixed lymphocyte reactions and elicit macrophage M2 polarization, mainly through TGF-β production [52]. Moreover, treatment with TLR3 and TLR4 agonists potentiates TGF-β and IL-6 secretion [53]. These characteristics make DFPCs promising candidates for the treatment of chronic inflammatory conditions.

## Tooth Germ Progenitor Cells

TGPCs were identified by Ikeda et al. in the dental mesenchyme of the third molar tooth germ during the late bell stage. TGPCs have been successfully transplanted in rat models of chronic hepatitis, preventing the progression of liver fibrosis and contributing to the normalization of liver function [54]. Thus far, little is known about TGPCs' immunomodulatory mechanisms.

## Gingiva-Derived Mesenchymal Stromal Cells

Zhang et al. [55] identified human gingiva-derived MSCs in 2009. GMSCs are easily isolated and rapidly expanded *ex vivo*, thus potentially representing an optimal source of MSCs in the clinical setting. GMSCs have been shown to efficiently inhibit T cell proliferation in response to mitogen stimulation and to induce IDO, IL-10, cyclooxygenase 2, and inducible nitric oxide synthase through IFN-γ secretion, thereby exerting a wide-ranging anti-inflammatory and immunomodulating action [56].

In animal models of contact hyper-reactivity and in an autoimmune arthritis model, systemic infusion of GMSCs attenuated pathological damage and suppressed Th17 activity, with a significant increase both in Tregs differentiation and IL-10 production [57]. Moreover, GMSCs elicited M2 polarization of macrophages and decreased Th17 cell expansion [58]. In addition, murine models of chemotherapy-induced oral mucositis showed significant clinical improvement after GMSC administration: in this setting, increased levels of manganese superoxide dismutase and hypoxia-inducible factors 1 and 2α were associated with lower rates of oxidative stress-induced apoptosis of epithelial cells [29].

## Additional Data on Dental Mesenchymal Stromal Cells and Other Cell Sources

Our paper focused on MSC interaction mainly with T cells and macrophages because most of the existing data on dental MSCs are restricted to these cell subtypes. However, MSCs in general have been deeply investigated for their interactions with other immune cells, with the majority of data coming from bone marrow MSCs [59].

MSCs notably inhibit the maturation of DCs and can promote plasmacytoid DC differentiation, with subsequent Th2 polarization of the immune response [60, 61]. Both PGE2 and IL-6 secretions have been postulated as possible mechanisms for DC modulation by MSCs [62, 63]. NK cells also interact with MSCs and are sometimes responsible for their death through cell lysis [64]. However, although only partially interfering with activated-NK activity, MSCs can block the proliferation of resting NKs. Moreover, MSCs prevent DC-mediated induction of T-cell effector functions, IDO and PGE2 being key mediators in this setting [65]. MSCs are also capable of B cell inhibition and can block antibody production [66]. Programmed-death-1 pathway, CCL2 production, and Blimp-1 inhibition seem to be responsible for this action [67, 68]. Finally, MSCs are demonstrated to promote the proliferation of CD4+ CD25+ FOXP3+ Tregs both *in vitro* and *in vivo* [69–71].

Although those data have not entirely been confirmed for dental MSCs, it is possible to hypothesize similar mechanisms underlying their immunomodulatory action. In fact, several comparative studies between dental MSCs and MSCs from other sources have been performed in the last years, but although significant differences were found in proliferative potential and both regenerative and differentiating properties, none of them was focused on immune-modulatory capabilities [72, 73].

## Discussion

MSCs have already been used as cell-based immunosuppressive therapies for various disorders, including neurologic, ocular, oral, cutaneous, cardiovascular, and autoimmune diseases [74, 75]. A growing number of clinical trials are using MSCs for therapeutic interventions in severe degenerative and inflammatory disorders. At the time of writing, almost more than 1,000 clinical trials were registered worldwide at ClinicalTrials.gov [4, 76], with MSCs becoming a powerful new tool for effective immunosuppression avoiding many unwanted adverse effects of conventional drugs [77]. Some types of dental MSCs have been shown to share both regenerative and immunoregulatory potentials, which are becoming extremely relevant for tissue engineering and regenerative medicine [78]. However, very few studies have explored their interactions with immune cells in any depth, and much less is known about the possible mechanisms of their activity. Taking into account the different types of MSCs isolated from teeth, including DPMSCs, MSCHEDTs, GMSCs, PDLMSCs, ABMSCs, DFPCs, and TGPCs, representing an easily accessible source of multipotent cells for clinical applications [79, 80], we are still far from a systematic investigation and comparative appreciation of their immunomodulatory properties [81].

One difficulty in such studies resides in the complexity of the stroma, whose tissue-resident cells interact in many ways with immune cells. The characterization of stromal subsets, which are often identified by combinations of markers that are not cell type-specific, has not been extensively carried out [82]. These subsets of mature stromal cells and MSCs from different tissues (bone marrow, adipose tissue, and umbilical cord, better studied so far) both promote active responses and suppress immune effector cells through regulatory circuits. Tissue stromal cells under inflammatory conditions drive the formation of immune cell aggregates, termed tertiary lymphoid structures [83], which disappear on a resolution of inflammation [84]. These structures actively drive inflammation, autoimmune responses, and autoantibody production, as well as promoting cancer progression, as prominently described in the lymph node stroma [85], but they also harbor tolerogenic potential, which depends on the inflammatory environment to be licensed [86].

Several mechanisms and molecules have been proposed for the immunoregulatory activity of MSCs in general, involving both cell contact and soluble mediators [87]. These have a protective role and stimulate growth and survival through paracrine secretion of bioactive molecules, collectively defined as the secretome. In many instances, the secretome has been shown to account for the effects of MSCs, so its exploitation may avoid the limitations associated with stem cell therapy [88, 89]. The secretome also contains extracellular vesicles (EVs) [90]. These released membrane vesicles, including exosomes, microparticles, microvesicles, and apoptotic bodies, can be regarded as a dynamic extracellular vesicular compartment, strategic for their paracrine or autocrine biological effects. They can contribute to tissue regeneration, but with their content rich in cytokines, chemokines, enzymes, growth factors, microRNAs, and other molecules, they may also be responsible for controlling interactions with immune cells, ensuring prevention of excessive tissue fibrosis, stimulation of angiogenesis, and immunomodulatory effects [91]. However, as for different sources of MSCs, and different culture and passage conditions, also for the secretome, differences in production protocols, cell source, and cellular age all impact its composition and anti-inflammatory action [92]. There is a need for focused mechanistic studies and standardized functional assays in the area of immunomodulation by MSCs because they are usually assessed by *in vitro* tests of inhibition of T lymphocyte proliferation, and only a few studies compare MSCs from different tissue sources, none at present with dental MSCs [93]. There is general agreement that pro-inflammatory environments are not permissive for endogenous stem and progenitor cells to initiate regenerative processes because stem and progenitor cells require a tolerogenic niche to survive and to promote repair and regeneration. MSCs from teeth have a central role in dampening inflammation locally as in periodontitis [94], and they achieve this effect through their secretome [93] and EVs. The cytokine content and immunoregulatory effect effects of the latter are variable across different diseases, so that the MSCs-EV fraction should be carefully evaluated in the context of the condition studied for the best therapeutic potential.

Although there are no direct studies of MSC interactions with neutrophils in tissues, MSCs inhibit neutrophil apoptosis, although they have no inhibitory effect on their phagocytic and chemotactic activity [95]. MSCs generally reduce the activation of innate immunity [92], and many of their effects are due to the secretion of IL-6, PGE2, and IL-17. Stromal cells also play a role in the induction of myeloid-derived suppressor cells, which can be a pathological differentiated type of neutrophil, in several conditions, including cancer, sepsis, and viral infections [96]. MSCs and their EVs have been shown to induce conversion of pro-inflammatory M1 into M2 macrophages, and EVs released by M2 macrophages can subsequently promote Treg formation [97–99]. MSCs also modulate immune cell function through inhibition of dendritic cell maturation and suppress the functions of T lymphocytes, B lymphocytes, and NK cells. Many reviews have appeared on this issue of immunosuppression related to clinical uses, for example [100–102].

Pulp-derived MSCs have been proposed for treating systemic disorders [87] and other types of MSCs, particularly in the area of neuroinflammatory and neurodegenerative diseases [103, 104], whereas EVs have been advocated for the control and therapy of autoimmunity [105]. This promising outlook is certainly reinforced by progress in transcriptomics and single-cell analysis of MSCs [106, 107], revealing different subsets and mechanisms of action. It is therefore not surprising that MSCs or their exosomes have recently been suggested as a treatment for severe COVID-19 [108–111]. This potential therapeutic strategy has been successfully used in a few reported cases [112] and is mainly based on the known immunomodulating actions of MSCs in acute respiratory infections, through induction of Tregs [113] and in their ability to counteract proinflammatory cytokines [114].

Recent studies have highlighted that MSCs aging may limit their function and therapeutic potential, with some evidence for reducing their immunosuppressive activity [115–117]. Senescent MSCs show decreased proliferative activity, smaller MSCs-EV size, and lower production of cytokines and chemokines; their ability to inhibit T cell proliferation is impaired while not suppressing NK, B lymphocytes, and macrophages [76, 118]. To address this issue, changes in the expression profiles (including transcriptomic, proteomic, epigenetic, and non-coding RNAs) of senescent MSCs have been explored, and some rejuvenation strategies devised, starting from the modulation of the microenvironment under hypoxic conditions [119, 120]. Data mining several genetic datasets, coupled with powerful bioinformatics applications, have revealed that upregulation of HLA class II antigen expression is central to the changes of aged MSCs, causing a pro-inflammatory phenotype and a decreased immunosuppressive function [121]. Again, little is known about replicative senescence (and its markers) and other effects of aging on dental MSCs.

The immunomodulatory properties of MSCs, at variance with other stem cells, contribute greatly to their therapeutic effects not only in immune-mediated diseases but also for the repair of tissue damages. This has been chiefly verified in several neurodegenerative disorders, such as Alzheimer's disease, amyotrophic lateral sclerosis, and Parkinson's disease, as well as in cerebrovascular damage and autoimmune disease as multiple sclerosis. The glia cells, activated in these conditions, constitute the main targets of the immunosuppressive action of MSCs [103]. Other cell types, predominantly macrophages, but also dendritic cells, induce a different inflammatory environment, in response to which MSCs display regulatory mechanisms tailored to the local situation and, thus, have been used for the treatment of various conditions such as graft-vs.-host disease [122], systemic lupus erythematosus [123], liver cirrhosis [124], and inflammatory bowel disease [125]. Their potential use in treating fibrotic and inflammatory diseases such as systemic sclerosis, chronic obstructive pulmonary disease, pulmonary fibrosis, and also severe asthma and COVID-19 should now be the logical next step forward.

These immunoregulatory properties and long-term stability of dental MSCs are of paramount importance for developing their application to autoimmune and other inflammatory conditions, as well as for continued renewal in regenerative medicine, as dampening inflammatory reactions promotes proliferation and differentiation of MSCs. Present data suggest that dental MSCs may be a useful source of MSCs for treating immune-mediated diseases.

However, the exact mechanisms responsible for dental MSC-mediated immunosuppression remain to be clarified. Moreover, it is not known whether dental MSCs' immunosuppressive function is impaired under local as well as systemic inflammatory conditions. This point is crucial to understanding whether autologous PDLC could be a reasonable source of MSCs for the treatment of autoimmune and other disorders. Despite the promising results achieved in dental MSCs and immunomodulation, this area of research needs to be methodically investigated.

## Data Availability Statement

The original contributions presented in the study are included in the article/supplementary material, further inquiries can be directed to the corresponding author.

## Author Contributions

RP and APa: conceptualization. FD and APi: literature revision and data curation. RP: supervision. APa, OT, and RP: writing—original draft preparation and writing—review and editing. All authors have read and agreed to the published version of the manuscript.

## Conflict of Interest

The authors declare that the research was conducted in the absence of any commercial or financial relationships that could be construed as a potential conflict of interest.
